# Addition of Allograft and Acromioclavicular Cerclage Improves Outcomes of Arthroscopic-Assisted Reconstruction of Acromioclavicular Separation With a Single Coracoclavicular Tunnel

**DOI:** 10.7759/cureus.28539

**Published:** 2022-08-29

**Authors:** Matthew P Noyes, Pablo Narbona, Paul C Brady, David P Huberty, Christopher R Adams, Javier Ardebol, Patrick J Denard

**Affiliations:** 1 Shoulder Surgery, Marion Health, Dayton, USA; 2 Shoulder Surgery and Arthroscopy, Sanatorio Allende, Córdoba, ARG; 3 Shoulder Surgery, Tennessee Orthopedic Clinics, Knoxville, USA; 4 Shoulder Surgery, Providence Willamette Falls Medical Center, Oregon City, USA; 5 Orthopedic Surgery, Naples Community Hospital, Naples, USA; 6 Shoulder Surgery, Oregon Shoulder Institute, Medford, USA

**Keywords:** reconstruction, suture tape, button, acromioclavicular, ac separation, arthroscopic

## Abstract

Objective

The purpose of this study was to prospectively evaluate the functional outcome and complications of unstable acromioclavicular (AC) joint separations repaired with a single coracoclavicular tunnel utilizing an arthroscopic-assisted curved button technique.

Methods

Thirty-five patients with a minimum of 12 months follow-up underwent arthroscopic-assisted AC joint reconstruction with suspensory button and 2 mm suture tape fixation using 3 mm tunnels. Functional outcome scores were analyzed preoperatively and at final follow-up with all complications noted.

Results

Comparing preoperative to postoperative values, all functional outcome scores improved. Three of the 16 (19%) patients that had a supplementary graft looped around the undersurface of the coracoid demonstrated loss of reduction compared to eight of the 19 (42%) that were treated with button and suture fixation alone (p = .138). No loss of reduction occurred in the subset of patients with AC joint supplementation. One (3%) patient sustained a distal clavicle fracture.

Conclusion

Arthroscopic-assisted AC joint reconstruction with a suspensory button construct demonstrates improved clinical outcomes with high patient satisfaction. While loss reduction remains problematic, smaller bone tunnels appear to lead to a low rate of iatrogenic fractures. The addition of a free tendon graft, as well as AC cerclage, appears to minimize loss of reduction.

## Introduction

A variety of techniques have been proposed for the surgical management of acromioclavicular (AC) joint separations. More than 60 techniques have been described to treat these challenging injuries [1]. In recent years, the trend has been toward anatomic reconstruction with bone tunnels. Additionally, arthroscopic-assisted techniques have been developed to limit the amount of dissection of the deltotrapezial fascia, improve visualization of the undersurface of the coracoid, and evaluate and treat associated glenohumeral joint pathology.

The two greatest challenges of AC joint reconstruction have been a postoperative loss of reduction and postoperative fracture of the clavicle or coracoid. Cook et al. reported an 80% rate of loss of reduction using the GraftRope (Arthex Inc., Naples, FL), which utilizes a 6 mm drill through both the clavicle and coracoid [2]. Similarly, Milewski et al. reported a loss of reduction in 50% and a 20% rate of iatrogenic coracoid fracture with the same single tunnel technique [3].

In an effort to decrease the risk of iatrogenic fracture and maintain reduction, new techniques have been developed [4]. One of these options uses curved buttons and suture tape placed through 2.4 to 3.0 mm bone tunnels. Theoretically, the smaller bone tunnels and broader surface area of the curved buttons may reduce the risk of iatrogenic bony fracture. However, there is limited information about the outcomes of this technique.

The purpose of this study was to prospectively evaluate the functional outcome and complication rate of unstable AC joint separations repaired with a single coracoclavicular tunnel utilizing an arthroscopic-assisted curved button technique. Our hypothesis was that the curved button technique would demonstrate improved functional outcomes in patients undergoing arthroscopic AC joint reconstruction with a low rate of iatrogenic bony fracture.

## Materials and methods

A prospective analysis was completed on arthroscopic-assisted AC reconstructions with the curved button construct performed at four institutions between January 2013 and December 2015. Institutional review board approval was obtained (Salus IRB, Austin, Texas, USA: Arthrex Inc., Protocol Number SOS #1) before commencing the study. Patients were recruited from clinic visits and offered the opportunity to participate in the study. Participation was voluntary and followed informed consent. They were informed that their participation was not mandatory and that treatment would not be impacted by participation.

Inclusion criteria included both acute and chronic high-grade III-V AC joint separations repaired with an arthroscopic-assisted technique during this time frame. AC joint separations were classified based on the Rockwood classification [5]. Acute injuries were defined as those treated within 4 weeks of injury, while chronic injuries were defined as those treated beyond 4 weeks from the time of injury. The minimum follow-up was 12 months for functional outcomes. Exclusion criteria were grade 1 and 2 acromioclavicular separations and revision reconstruction procedures.

Functional status was determined by a review of prospectively collected data preoperatively and at the final follow-up. American Shoulder Elbow Society (ASES) scores [6] and Simple Shoulder Test (SST) were recorded. Pre and post-operative pain were graded from 0 to 10 on a visual analog scale (VAS). Additional procedures and all complications were reported. To standardize measurements, preoperative coracoclavicular (CC) distances were measured on bilateral Zanca view radiographs with a straight line from the most superior aspect of the coracoid to the undersurface of the clavicle. In addition, magnetic resonance imaging was obtained on all patients undergoing the surgical procedure to assess for concomitant lesions [7,8]. A postoperative Zanca view was obtained immediately postoperatively and at final follow-up. We defined loss of reduction as a greater than or equal to 50% increase in the CC distance at the final follow-up compared to immediately postoperative [9]. Postoperative radiographs were also reviewed for the presence or absence of clavicular tunnel widening.

Surgical technique

Patients were placed in either the beach chair or lateral decubitus position based on surgeon preference. A 4-cm superior incision was made over the clavicle, extending laterally to the AC joint. Periosteal flaps were raised and temporary reduction of the AC joint was performed with a 2 mm K-wire. Next, a standard diagnostic viewing portal was established. An arthroscopic radiofrequency ablator was used to make a window in the rotator interval to identify the coracoid. A 70° arthroscope was used to clearly visualize the undersurface of the coracoid as well as the medial and lateral margins to place a drill hole in the center of the base of the coracoid process.

**Figure 1:**
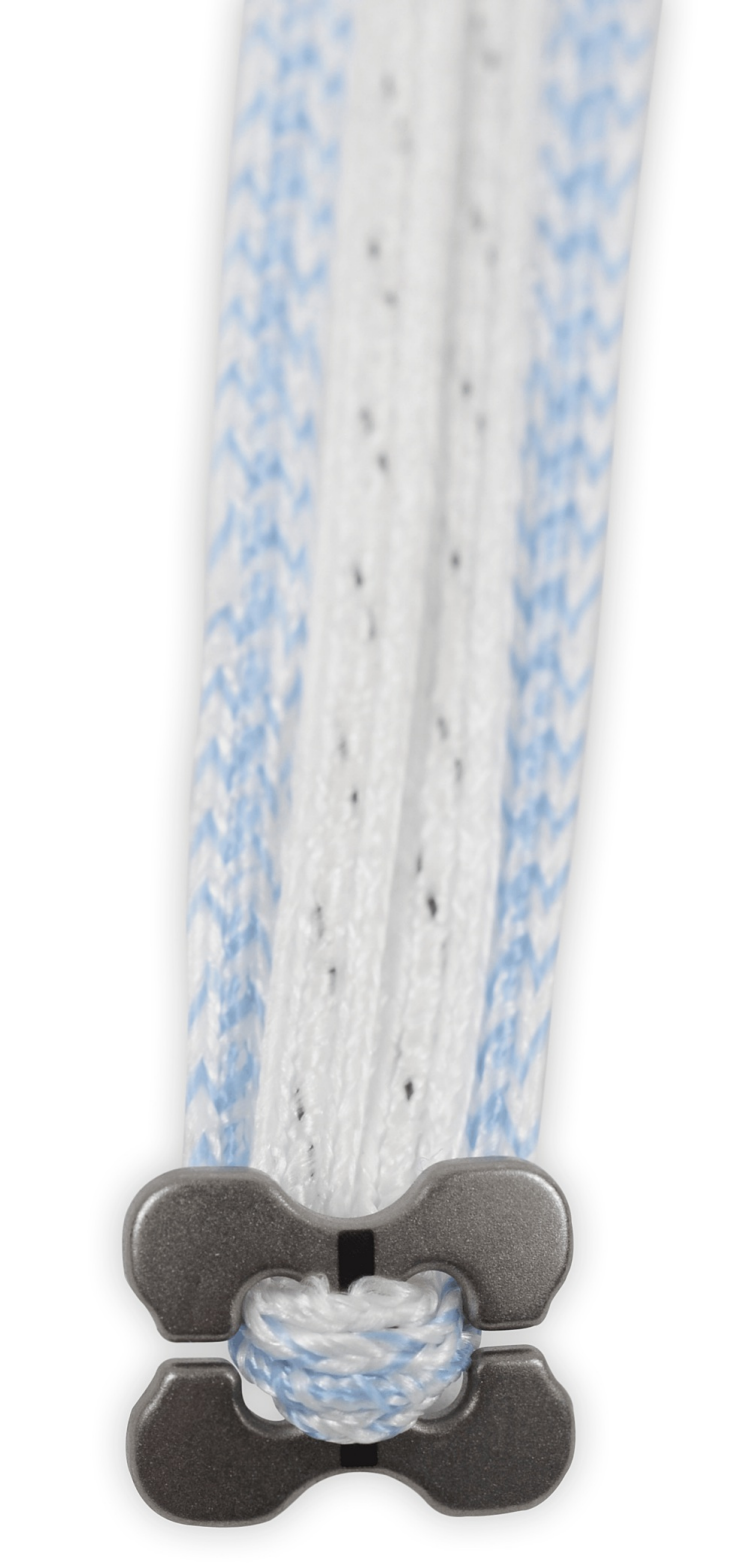
Curved button and 2 mm suture tape used for fixation

An outside-in technique was used to establish a mid-anterior portal and a flexible cannula (PassPort cannula, Arthrex, Inc., Naples, Florida) was placed through the rotator interval. An adjustable C-shaped guide was introduced into the subcoracoid space through the anterior portal. The clavicle drill hole was placed approximately 35 mm from the lateral clavicle margin, splitting the difference between the conoid and trapezoid drill tunnels as reported by Carofino et al. [10]. After the aiming sleeve was aligned appropriately on the undersurface of the coracoid, a cannulated drill bit was used to drill bicortically through the clavicle and coracoid. During the study period, a 3.0 mm drill bit was used in all cases. A nitinol wire was passed through the cannulated drill bit and retrieved out the anterior portal. A #2 suture with a closed loop on one end (FiberLink; Arthrex, Inc.) was then shuttled out through the clavicle. A curved button (Dog Bone; Arthrex, Inc) was preloaded with two 2 mm suture tapes (Figure 1). The loop suture was then used to shuttle the preloaded button in a retrograde fashion through the anterior portal until it rested on the undersurface of the coracoid. The position of the button was confirmed arthroscopically. A second button was then loaded onto the free ends of the suture tape exiting the clavicle. The sutures were tied to secure the construct.

During the study period, it was most common for the surgeons to use isolated suture tape and button suspensory fixation for acute injuries or those under 4 weeks from the time of injury. In some cases of acute injuries (<4 weeks from injury), the fixation was supplemented with semitendinosus allograft based on surgeon preference. For all chronic cases (> 4 weeks from injury), a semitendinosus allograft was utilized to supplement fixation. The graft was whip stitched on both ends with a # 2 high strength suture and was shuttled through the anterior portal and looped under the coracoid. The graft was retrieved from the medial aspect of the coracoid posterior to the clavicle and the lateral aspect was retrieved anterior to the clavicle. The graft was then sutured together over the superior aspect of the clavicle with the # 2 suture.

In addition, an AC joint cerclage technique was utilized in a subset of patients. A 2.0 mm drill bit was used to drill bicortically from posterior to anterior in the distal clavicle and acromion. A # 5 high-strength suture was then used to create a figure-of-eight stitch over the AC joint, shuttling the suture through the distal clavicle from posterior to anterior and then the acromion in the same fashion. The suture limbs were then tied completing the cerclage.

Postoperatively, patients were immobilized in a sling for 6 weeks, allowing passive external rotation at the side as tolerated. After 6 weeks, the sling was discontinued and passive forward elevation and table slides were allowed. At three months postoperative, strengthening was initiated. Concomitant procedures included six (17%) acromioclavicular joint cerclages with a # 5 suture, five (14%) distal clavicle excisions, two (6%) SLAP (Superior Labrum from Anterior to Posterior) repairs, two (6%) arthroscopic rotator cuff repairs, two (6%) arthroscopic biceps tenodeses, one (3%) arthroscopic posterior labral repair, and one (3%) bony Bankart repair.

Statistical analysis

Continuous data were described by mean and standard deviation. A t-test was used to analyze the difference in pre and post-outcome scores for forward flexion, VAS score, ASES score, and SST score. Proportional differences were compared using a chi-square test. Two-tailed P <.05 was considered significant. All data are included in this manuscript and available upon request.

## Results

Forty-six reconstructions were performed during the study period. Eleven patients were lost to follow-up and did not complete the one-year follow-up requirement, leaving 35 (76%) patients available for analysis. The mean age of the cohort was 39.3 years (range 16-67) at the time of surgery. Thirty-two patients (91%) were men and 23 (66%) involved the dominant extremity. The mean follow-up was 12.7 months. Twenty-two were grade III injuries, two were grade IV, and 11 were grade V. Injuries were caused by sports in 13 patients, a fall in 12 patients, and a motor vehicle accident in 10 patients. Twenty-two were acute injuries while 13 were chronic injuries with symptoms reported for a mean of 23 months (range, 12-60 weeks). All 13 patients with chronic AC joint separations received a semitendinosus allograft supplementation. Of the 22 patients with acute injuries, three received semitendinosus allografts for additional fixation. The operative characteristics are summarized in Table 1.

**Table 1:** Operative characteristics of arthroscopic-assisted suture button technique Abbreviations: DBB, dog-bone button, CC, coracoclavicular; AC acromioclavicular a Data correspond to the Rockwood classification

DBB Reconstructions (n=35)	No.	SD	%
Age of patient (mean, SD)	39.9	14.2	
Male sex (n, %)	32		91%
Dominant Arm (n, %)	23		66%
Injury Grade ^a ^(n, %):			
Grade III	22		63%
Grade IV	2		6%
Grade V	11		31%
Graft Supplementation (n, %)	16		46%
AC cerclage (n, %)	6		17%
Preoperative CC distance mm (mean, SD)	21.4	4.9	
Contralateral (unaffected) CC distance mm (mean, SD)	9.2	2.2	

Comparing preoperative to postoperative values, ASES scores improved from 44.6 to 86.6 (P < .001) and the SST score improved from 6.5 to 11.3 (P < .001). The mean VAS pain score improved from 7.1 to 1.8 (P < .001). Eleven patients (31%) lost reduction, of which 8 were acute injuries (8 of 22; 36%) and 3 were chronic injuries (3 of 13; 23%). The mean CC distance on the operative side was 21.4 + 4.9 mm (range 15.0 - 35.8 mm) preoperatively compared with a mean of 9.2 + 2.2 mm (range 5.9 - 14.2 mm) on the contralateral side. At the final postoperative follow-up, the mean CC distance on the operative side significantly improved to 11.4 + 3.7 mm (range 5.0 - 17.2 mm) (P < .001). The operative results are summarized in Table 2.

**Table 2:** Operative results of entire cohort undergoing arthroscopic-assisted suture button technique Abbreviations: ASES, American Shoulder Elbow Society; SST, Simple Shoulder Test; VAS, Visual Analog Score

	Preoperative		Postoperative		
	Mean	SD	Mean	SD	P Value
ASES score	44.6	13.5	86.6	6.8	P= < .001
SST score	6.5	2.7	11.3	1.5	P= < .001
VAS pain score	7.1	1.8	1.8	1.1	P= < .001

Results were also stratified by the surgical technique. Three of the 16 (19%) patients that had a supplementary graft looped around the undersurface of the coracoid demonstrated loss of reduction compared to eight of the 19 (42%) that were treated with an isolated dog-bone button (DBB) and suture (P = .138). Comparing preoperative to postoperative values in the supplementary graft group, ASES scores improved from 45.6 to 87.1 (P < .001), and the SST score improved from 5.3 to 11.1 (P < .001). The mean VAS pain score improved from 5.9 to 1.0 (P < .001). The non-graft group's preoperative ASES score was 42.7 and postoperatively, it was 87.9 (P < .001). The SST score improved from 5.0 to 10.9 (P < .001) while the mean VAS score decreased from 6.5 to 1.9 (P <.001). The supplementary graft group mean CC distance preoperatively was 20.8 + 5.1 mm (range 17.3 - 35.8 mm), and at final follow-up, the CC distance significantly improved to 10.0 + 2.8 mm (range 7.0 - 17.2 mm) (P < .001). The group without additional graft mean CC distance preoperatively was 22.2 + 4.7 mm (range 15 - 29.5 mm), and at final follow-up, it improved to 11.9 + 3.7 mm (range 5-17 mm) (P < .001). Stratified surgical results are summarized in Table 3. Additionally, six (17%) patients (all of whom had a loop graft) also had an AC joint cerclage with a # 5 suture. No loss of reduction occurred in the subset of patients that had AC joint supplementation compared to 38% in the group without AC joint supplementation (P = .065).

**Table 3:** Functional outcomes of cohorts based on reduction of the AC joint Abbreviations: ASES, American Shoulder Elbow Surgeons; SST, Simple Shoulder Test; VAS, visual analog score; CC, coracoclavicular; AC, acromioclavicular

	Maintained Reduction		Lost Reduction		
	Mean	SD	Mean	SD	P Value
ASES score	88.8	7.2	84.5	6.2	P = .09
SST score	11.5	0.9	9.5	2.3	P= < .001
SSV score	91.3	8.2	79.6	9.6	P= < .001
VAS pain score	0.6	0.5	1.7	1.0	P = .005
CC distance	9.3	2.2	16.6	2.1	P= < .001

**Figure 2:**
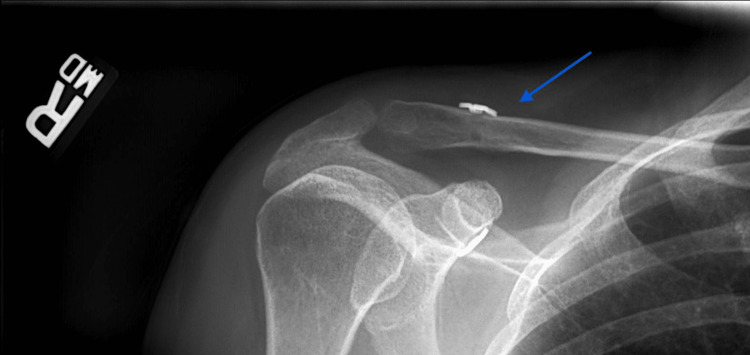
Immediate postoperative radiograph of a right shoulder demonstrates acromioclavicular reconstruction (blue arrow) with a single coracoclavicular tunnel

**Figure 3:**
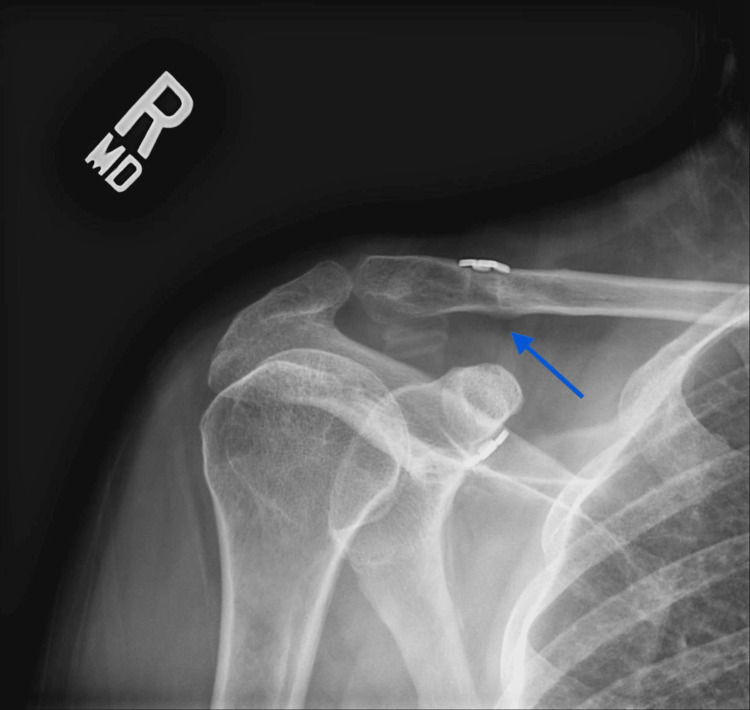
Twelve-month postoperative radiograph demonstrates significant tunnel widening (blue arrow) in the clavicle

Twenty patients (57%) had evidence of tunnel widening on the final follow-up radiographs (Figures 2, 3). Tunnel widening occurred in 9 patients (56%) that received graft supplementation versus 11 patients (58%) that did not receive an additional graft (p = .765). There was no evidence of tunnel widening in the 6 patients that received AC cerclage supplementation compared to 69% in the 29 patients that did not have AC joint supplementation (p = .002). 

Overall, 30 patients (86%) reported being satisfied with the procedure and 31 patients (89%) returned to their previous activity or work level. No secondary procedures were performed, and there were no instances of deep wound infection or implant failure in the cohort. Complications included one (3%) distal clavicle fracture and one (3%) case of postoperative stiffness. There were no postoperative coracoid fractures.

## Discussion

This study attempted to determine the ability of a curved button construct with suture tape and a single tunnel for unstable AC joint separations to improve functional outcomes and minimize complications, specifically loss of reduction and fracture. The data support the study's hypothesis that a curved button construct improves functional outcomes in patients with AC joint separations. Outcomes in our study were favorable when compared with previous reports of arthroscopic-assisted AC joint reconstruction. More importantly, there was no evidence of coracoid facture from either the small drill bit used for coracoid drilling or looping the graft around the coracoid.

A variety of surgical techniques have been proposed for the treatment of AC joint separations. Classically, the Weaver-Dunn procedure involved the excision of the distal clavicle and the transfer of the coracoacromial ligament to the distal end of the clavicle as a substitute for the disrupted CC ligaments [11]. However, the strength of this procedure is only 25% of the native CC ligaments and the failure rate is as high as 30% [12]. Subsequent authors modified the technique to a more anatomic approach in order to improve initial strength and more closely mimic native translation. Carofino et al. reported an anatomic open CC ligament reconstructive procedure using free tendon allograft in 17 patients [10]. The mean ASES score improved from 52 preoperative to 92 at the final follow-up, with an 18% failure rate. While their open procedure required dissection of the deltotrapezial fascia, the free tendon graft was looped around the coracoid, eliminating any drill holes that may propagate to cause a fracture.

Arthroscopic-assisted AC reconstruction has recently gained popularity as a way to improve visualization of the undersurface of the coracoid and treat intraarticular pathology. However, initial techniques required larger diameter drill holes to be placed into the coracoid and clavicle, which resulted in a high rate of iatrogenic fracture. Cook et al. reported on AC joint reconstructions using the GraftRope, which used 6 mm tunnels in the clavicle and coracoid [2]. In their series, 80% of the patients lost reduction and only 40% were able to return to full duty in a military population. In a similar technique, Coale et al. reported postoperative coracoid fractures in 91.3% of cases [13]. They concluded that the transclavicular-transcoracoid techniques cannot restore CC footprints anatomically without a significant risk of coracoid fracture.

**Figure 4:**
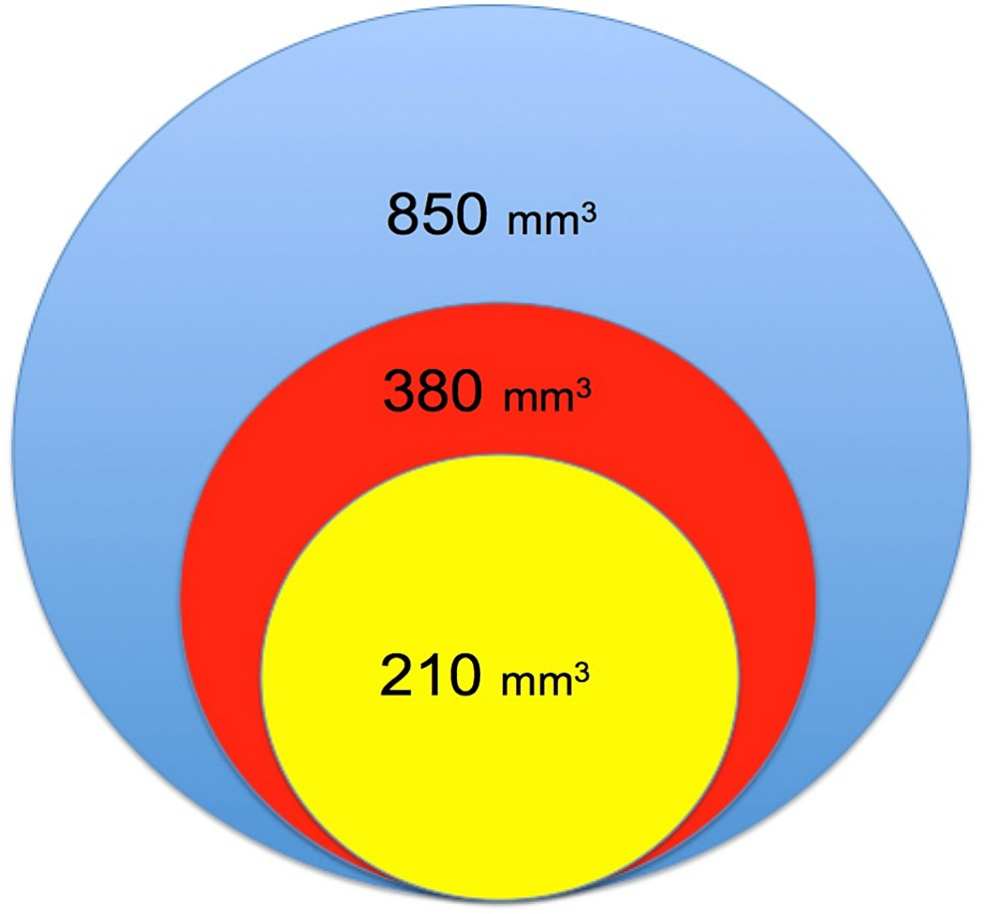
Schematic demonstrating the volume of bone removal from coracoid and clavicle tunnel drilling in acromioclavicular joint reconstruction 850 mm^3^ = 6 mm drill bit, 380 mm^3^ = 4mm drill bit and 210 mm^3^ = 3 mm drill bit. V = volume of bone loss, r = radius of drill tunnel, and h = depth of coracoid and clavicle

In contrast to the aforementioned studies, in the current study using 3 mm tunnels, we did not observe any coracoid fractures and only one patient sustained a clavicle fracture. Martetschlager et al. performed a biomechanical study evaluating different drill hole sizes and configurations in the coracoid process [14]. They reported that 4.0 mm drill holes failed by coracoid fracture, whereas the use of 2.4 mm drill holes transferred the mode of failure to the conjoint tendon (indicating a low risk of bony fracture). These results reflect the decreased volume of bone removal with smaller drill holes. The volume of bone removed between different size drill bits can be estimated using the formula V=πr2h. For example, based on an estimated depth of 15 mm for the coracoid and clavicle, a 3.0 mm drill bit results in four times less bone removed than a 6.0 mm drill bit (212 mm^3^ vs. 848 mm^3^) (Figure 4). We believe the low rate of iatrogenic fracture seen in the current study is a reflection of this decreased bone removal compared to previous techniques.

In addition to smaller bone tunnels, the current study may have other technical implications including graft technique, the timing of the graft, and AC joint cerclage. Milewski et al. reported an 80% failure rate in a group of 10 patients augmented with grafts passed through a coracoid tunnel [3]. In contrast, Tomlinson et al. reported an 80% rate of maintenance of reduction 5 months postoperatively in acute and chronic separations managed with a loop technique [15]. VanSice and Savoie reported that 11 of 12 patients (92%) treated with a loop technique maintained reduction [16]. The current study provides further support that a loop technique with a graft is safe and efficacious. As opposed to creating large coracoid tunnels, the loop technique for graft augmentation preserves bone thereby lowering the risk of coracoid fracture.

Based on our observations, we believe a graft should be performed in all cases using a single coracoclavicular tunnel technique. In the acute cases, we initially used the curved button alone without graft. While this provides exceptional superior-inferior stability, several of our patients lost reduction with this isolated technique despite being performed within 4 weeks of injury. In addition to biologic support, the graft likely provides additional rotational and anterior-posterior stability to the construct. This is reflected in our lower rate of loss of reduction in graft cases (19%) compared to that in which an isolated curved button with a suture was used (42%).

While an AC separation involves disruption to both the AC and CC ligaments, much of the surgical focus has been on the reconstruction of the CC ligaments alone. However, there is some evidence that supplemental fixation of the AC joint is valuable. In a biomechanical study, Ladermann et al. demonstrated that cerclages around the CC and AC joints more closely mimicked native stresses compared to a plating technique or a dual button technique [17]. In a clinical study on the same cerclage technique, 76% of patients maintained the reduction to within 5 mm of the opposite side [18]. Interestingly, in the current study, all six patients who had supplemental AC joint fixation maintained reduction. Furthermore, it is notable that none of our patients with supplemental AC joint fixation had postoperative clavicular tunnel widening. Since tunnel widening reflects the motion of the reconstruction, the lack of tunnel widening with supplemental AC joint fixation provides clinical evidence of improved stability with this technique. While our numbers are small, we believe our high success rate with this additional technique and the absence of tunnel widening supports the use of supplemental AC joint fixation, and we currently use it in all cases.

This study has several limitations. First, the curved button and suture tape construct was not compared with another treatment group or nonoperative treatment. Our goal was simply to evaluate the functional outcomes and complications of the technique. Second, our minimum follow-up was only 12 months, which could bias the complication rate reported. However, the majority of failures occur radiographically within 6 months, with one paper showing high failure rates as early as an average of 7 weeks [2]. Finally, we had a small cohort size, particularly with regard to the number of patients that had supplemental AC joint cerclage fixation with # 5 FiberWire. Further study is needed to confirm our findings with regard to supplemental AC joint fixation.

## Conclusions

Arthroscopic-assisted AC joint reconstruction with a suspensory button construct demonstrates improved clinical outcomes with high patient satisfaction. While reduction loss remains problematic, smaller bone tunnels and larger curved button surface areas appear to lead to a lower rate of iatrogenic fractures. Using the arthroscope can also prevent further damage to surrounding tissues while allowing direct visualization. The addition of a free tendon graft as well as AC cerclage appear to minimize loss of reduction. As a result, AC reconstruction with dual fixation may provide better outcomes than single CC fixation alone. Regardless of these positive findings, additional studies are needed.
